# Energy scavenging based on a single-crystal PMN-PT nanobelt

**DOI:** 10.1038/srep22513

**Published:** 2016-03-01

**Authors:** Fan Wu, Wei Cai, Yao-Wen Yeh, Shiyou Xu, Nan Yao

**Affiliations:** 1 Princeton Institute for the Science and Technology of Materials (PRISM), Princeton University, 70 Prospect Avenue, Princeton, New Jersey 08544, USA

## Abstract

Self-powered nanodevices scavenging mechanical energy require piezoelectric nanostructures with high piezoelectric coefficients. Here we report the fabrication of a single-crystal (1 − x)Pb(Mg_1/3_Nb_2/3_)O_3_ − xPbTiO_3_ (PMN-PT) nanobelt with a superior piezoelectric constant (d_33_ = ~550 pm/V), which is approximately ~150%, 430%, and 2100% of the largest reported values for previous PMN-PT, PZT and ZnO nanostructures, respectively. The high d_33_ of the single-crystalline PMN-PT nanobelt results from the precise orientation control during its fabrication. As a demonstration of its application in energy scavenging, a piezoelectric nanogenerator (PNG) is built on the single PMN-PT nanobelt, generating a maximum output voltage of ~1.2 V. This value is ~4 times higher than that of a single-CdTe PNG, ~13 times higher than that of a single-ZnSnO_3_ PNG, and ~26 times higher than that of a single-ZnO PNG. The profoundly increased output voltage of a lateral PNG built on a single PMN-PT nanobelt demonstrates the potential application of PMN-PT nanostructures in energy harvesting, thus enriching the material choices for PNGs.

Piezoelectric nanogenerators (PNG) have attracted extensive attention because they can harvest ubiquitous mechanical energies from ambient environment at any time^1^, for self-powered nanosystems that enable continuous operations of implantable biodevices, micro-electrochemical systems, wireless sensors and portable/wearable electronics, without the trouble of charging and replacing power sources^2^. At first, PNGs were based on vertically aligned nanowires of different materials, including ZnO^3^, AlGaN^1^, GaN^1^,^4^,^5^, InN^1^,^6^, PZT^7^, and CdS^8^. The rubbing of the electrode and the nanowire in vertical configuration results in mechanical instability, which is undesirable and avoided by the creation of a “lateral PNG”^9^ in 2009, using a lateral piezoelectric nanowire with both ends fixed to a flexible substrate. Since then, various materials have been selected for such lateral PNGs, including ZnO^9^,^10^, ZnSnO_3_^11^ and CdTe^12^ nano/microwires. However, the piezoelectric coefficients (d_33_) of these materials are not high enough (e.g. 12.4 pm/V for ZnO^13^, and 11.06 pm/V for ZnSnO_3_^14^), resulting to small output voltages and limited application of lateral PNGs based on a single nanostructure. Therefore the major challenge is to find alternative materials with higher piezoelectric coefficients for lateral PNGs, such that their output voltage can be profoundly increased^15^.

Among the intensively studied piezoelectric materials, relaxor ferroelectric (1 −x)Pb (Mg_1/3_Nb_2/3_)O_3_ −x PbTiO_3_ (PMN-PT) is highly desirable as a next-generation piezoelectric material due to its excellent piezoelectric properties (a d_33_ up to 2500 pm/V^16^ for PMN-PT bulk). The difficulty of fabricating PMN-PT nanostructures with excellent piezoelectric performances, readily available for PNG device assembling lies in the fact that the piezoelectric properties of PMN-PT will be weakened by up to 92%^17^ if deviated from the optimum composition and orientation. The first synthesis of 1-D PMN-PT nanostructures was reported in 2012 by Xu *et al.*^15^, who successfully used a bottom-up (hydrothermal) method to obtain single-crystal piezoelectric PMN-PT nanowires. However, the composition and orientation of each individual PMN-PT nanowire can not be precisely controlled by that bottom-up approach^15^, making it impractical to guarantee the excellent piezoelectric performance of each individual PMN-PT nanowire. For the same reason, a lateral PNG based on a single PMN-PT nanostructure, though theoretically promising for a high output voltage, has never been realized.

In this paper, we report the unprecedented fabrication of a single-crystal PMN-PT nanobelt by a top-down method. The crystal orientation, dimension and composition of the PMN-PT nanobelt are precisely controlled during fabrication to enhance the piezoelectric properties. The resultant d_33_ of the obtained nanobelt reaches ~550 pm/V, which is ~150%, 430%, and 2100% of the largest reported values for previous PMN-PT^15^, PZT^18^, and ZnO^19^ nanostructures. A lateral PNG based on a single PMN-PT nanobelt is designed and built to demonstrate the PMN-PT nanobelt’s efficient application in harvesting mechanical energies. During transportation and device assembling, the nanobelt’s orientation is carefully controlled. The output voltage of the single-PMN-PT- nanobelt PNG reaches 1.2 V, which is ~4 times higher than that of a single-CdTe PNG^12^, ~13 times higher than that of a single-ZnSnO_3_ PNG^11^, and ~26 times higher than that of a single-ZnO PNG^9^,^10^. The study herein shows the first PMN-PT nanobelt readily available for piezoelectric energy harvesting with a high piezoelectric coefficient, due to the precise control over the orientation during fabrication, transportation and device assembling processes. The lateral PNG based on a single PMN-PT nanostructure generates a profoundly increased output voltage, expanding the material choices and practical applications of such PNGs in wearable/portable devices and flexible self-powered electronic devices.

## Results and Discussions

**Figure 1:**
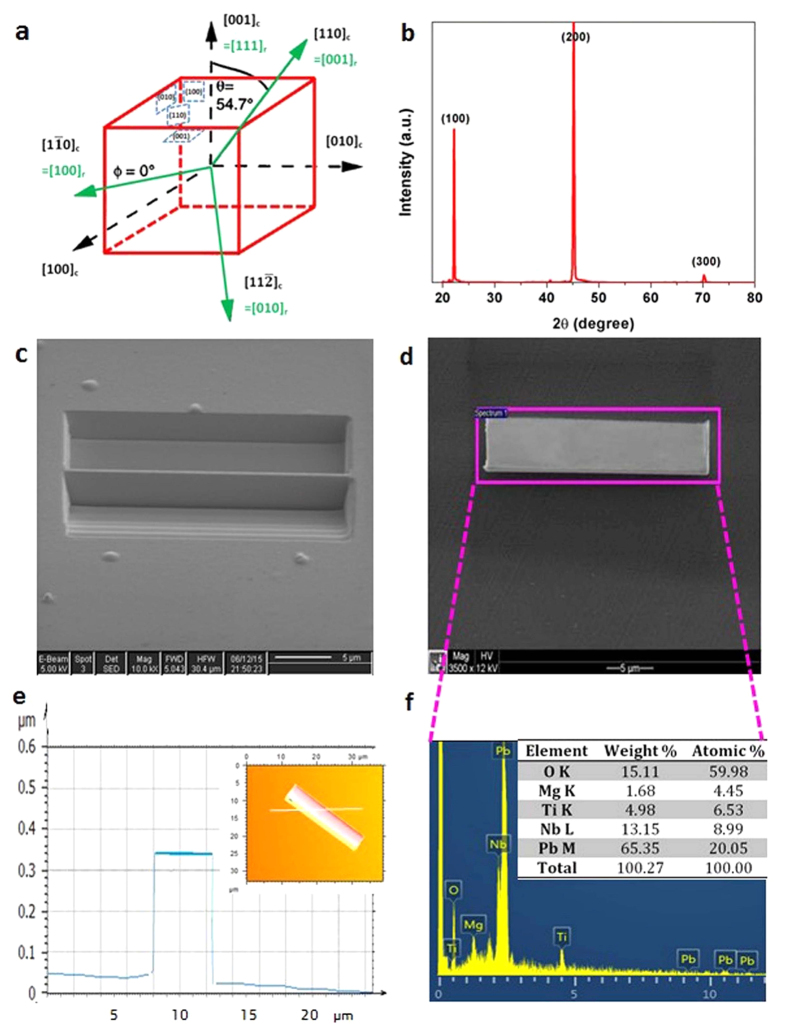
PMN-PT nanobelt preparation and preliminary characterization. (**a**) Schematic demonstration of the PMN-PT crystal lattice with labelled crystal directions in both rhombohedral coordinate system and cubic coordinate system. (**b**) Room temperature X-ray θ–2 θ diffraction pattern of the as-received PMN–PT substrate. (**c**) The FIB cutting process of a (001)_c_ surface dominated nanobelt. (**d**) SEM image showing the morphology of the FIB-cut (001)_c_ surface dominated PMN-PT nanobelt, which has a well-defined rectangular shape with sharp contours (**e**) Inset: the 3D topographical map of FIB-cut (001)_c_ surface dominated PMN-PT nanobelt by confocal microscopy; Main figure: the corresponding line profile derived from the inset. (**f**) The energy dispersive spectrum (EDS) generated from the pink box in panel (**d**) and quantitative summary.

The PMN-PT nanobelt was prepared using the Focused Ion Beam (FIB) technique. By carefully aligning the bulk PMN-PT crystal surface with respect to the incident ion beam during FIB cutting, nanostructures with any desirable out-of-plane orientations (e.g. <100>_c_, <110>_c_ and <111>_c_) and shapes (e.g. nanorods, nanowires and nanobelts) can be obtained. For readers to clearly understand the crystal orientations within PMN-PT crystal lattice, the crystal directions are schematically illustrated in Fig. 1a in both rhombohedral coordinate system (solid green lines with subscripts r) and cubic coordinate system (dashed black lines subscripts c). PMN-PT exhibits its best piezoelectric performance along [001]_c_ direction, with the composition at morphotropic phase boundary (MPB) region (Supplementary information section 3). If deviated from [001]_c_ direction, the piezoelectric performance may be weakened by up to 92%^17^. Therefore the crystal orientation of PMN-PT bulk has to be examined before FIB-cutting. Figure 1b shows the X-ray diffraction (XRD) θ–2θ pattern of the PMN-PT bulk crystal, in which only (100), (200), and (300) peaks were observed, revealing not only the single-crystalline nature but also the out-of-plane orientation ({001}_c_) of the bulk crystal. Figure 1c shows the FIB-cutting of (001)_c_ surface dominated PMN-PT nanobelt (hereafter “001NB”). Specifically, two big holes were dug by the ion beam besides a rectangular region of ~25 μm long and <1 μm wide, which was further thinned down by small-current ion beam (50 pA) to <0.3 μm wide. Then the thin lamella was cut free from the bulk crystal and became the 001NB. It is worth to note that FIB is also a flexible and convenient tool to prepare PMN-PT nanostructures with any orientation (e.g. (011)_c_ nanobelts) or shape (e.g. (001)_c_ nanorod), as shown in supplementary information Figure S1a and b.

The FIB prepared 001NB was lifted out for subsequent characterization and device fabrication. Figure 1d shows the SEM image of the 001NB, which has a well-defined rectangular shape with dimensions of 24 μm long and 5 μm wide. The thickness of the nanobelt was measured by 3D confocal microscopy as shown in Fig. 1e. According to the height profile derived from the 3D topographical map (inset), the average thickness of the nanobelt is ~250 nm. Thus the width-to-height and length-to-width ratios are obtained as ~20:1 and 4:1, respectively. The MPB composition of 001NB was confirmed by energy dispersive spectroscopy (EDS) as shown in Fig. 1f. The quantitative analysis of the elemental composition is summarized in the inset. The atomic ratio between Nb (8.99%) and Mg (4.45%) is ~2.02, close to the stoichiometric ratio of 2. The atomic ratio between PMN and PT is ~2.05, indicating a compositional formula of 0.67 Pb(Mg_1/3_Nb_2/3_)O_3_–0.33 PbTiO_3_ for the 001NB. This composition falls within the MPB region (x = 0.30–0.35) for PMN-PT with superior piezoelectric properties (supplementary information section 3.1). The confocal microscopy and SEM analyses of (011)_c_ nanobelt and (001)_c_ nanorod were also performed and shown in supplementary information Figure S2-5.

**Figure 2:**
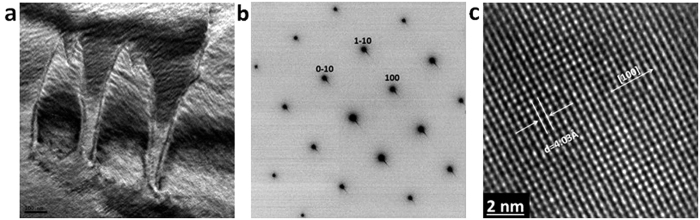
Microstructural characterization of PMN-PT nanobelt by TEM. (**a**) A typical bright-field TEM image showing the narrow strip-like domain patterns in the FIB-cut (001)_c_ surface dominated PMNPT nanobelt. (**b**) The [001] selected area electron diffraction (SAED) pattern of the FIB-cut (001)_c_ surface dominated PMNPT nanobelt. (**c**) A high-resolution TEM image demonstrating a defect-free single-crystalline structure of the nanobelt. The 4.03 Å lattice spacing corresponds to the (001)_c_ lattice plane.

The detailed micro and nano-structural characterizations were performed by TEM. The typical bright-field TEM image (Fig. 2a) shows the narrow strip-like domain patterns, representing antiparallel domains. The domain pattern of PMN–PT nanobelt results from the random field (induced by built-in charge disorder) in PMN-type relaxors, revealing the spatial inhomogeneity of ferroelectric domain structures. The interaction between nanopolar clusters and random internal field determines the ferroelectric domain state in relaxor-type ferroelectrics. The selected area electron diffraction (SAED) pattern along [001]_c_ direction is obtained from a large area and shown in Fig. 2b, indicating a uniform single crystalline structure for the PMN–PT nanobelt. This is further proved by a high-resolution TEM image (Fig. 2c), demonstrating a defect-free single-crystalline structure. The 4.03 Å lattice spacing corresponds to the (100)_c_ lattice plane and is labelled on the image.

**Figure 3:**
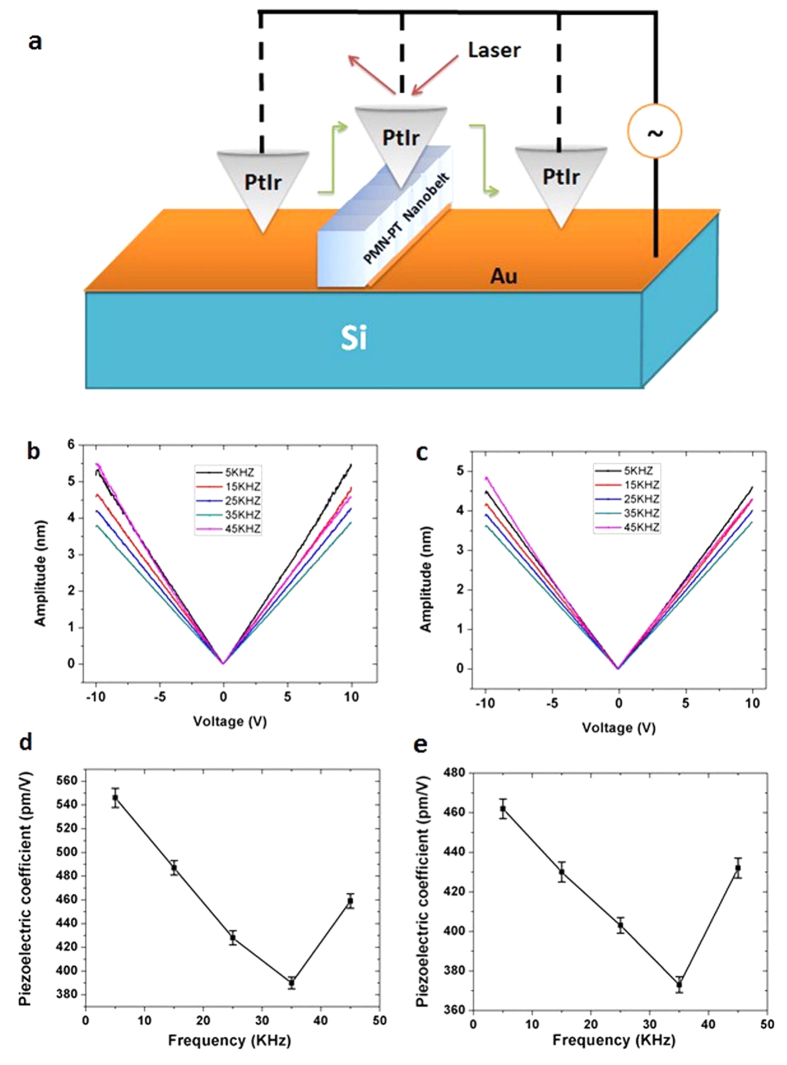
PFM characterization of PMN-PT nanobelt (001NB). (**a**) The schematic illustration of the experimental PFM characterization setup of the 001NB. (**b,c**) The typical piezoelectric displacement vs voltage curves from two different positions on 001NB. (**d,e**) The frequency dependences of the piezoelectric coefficients measured at the two different positions.

To study the piezoelectric properties of the 001NB, Piezo-response Force Microscopy (PFM) was used, enabling the characterization of piezoelectric properties on nanoscale with high resolution^20^ (supplementary information section 7). The PFM characterization setup is illustrated in Fig. 3a. Specifically, the tip was scanned above the PMN-PT nanobelt, and the responsive piezoelectric strain in the nanobelt caused the displacement of the cantilever, along with a phase shift between the reference and input signals. It is worth to note that the lock-in amplifier in PFM separates the signal from random noise, retrieving the amplitude and phase of surface deformation induced by the converse piezoelectric effect. The final output signal is only relevant to the amplitudes of the reference signal and the signal induced by piezoelectric sample, as well as the phase shift between the two signals. The deformation of tip (which was a constant during PFM measurements because of the same set-point value used during experiments) is not involved or reflected in the final output signal. In other words, the lock-in amplifier corrected the deformation of the tip involved in the PFM measurements. The simultaneously obtained atomic force microscope (AFM) morphology map and PFM phase map demonstrate that the 001NB has an obvious piezoelectric response (supplementary information Figure S6a and b). Therefore it is attractive to further characterize the piezoelectric property of 001NB quantitatively.

To measure the piezoelectric coefficient of 001NB, piezoelectric displacement vs. voltage curves were obtained and shown in Fig. 3b,c. The effective piezoelectric coefficients d_33_ are derived from the slopes of those curves and summarized in Table 1. The maximum obtained effective piezoelectric coefficient d_33_ is 550 pm/V, with a statistically averaged value of 448.4 pm/V. The maximum measured d_33_ value here is ~2100% and 430% of the maximum reported values of 1-D ZnO (26.7 pm/V^19^) and 1-D PZT (130 pm/V^18^) nanostructures, respectively. Moreover, this value is also ~150% of the value (373 pm/V) reported for PMN-PT nanowires^15^, which was the ground-breaking 1-D PMN-PT nanostructure having the highest reported piezoelectric constant^15^. The record-high d_33_ results from the precise control of the nanobelt’s crystal orientation not only during fabrication, but also during the set-up and manipulation for PFM characterization, which is unfulfillable for other methods (supplementary information section 4).

To ensure the reliability and accuracy of the PFM measurement and rule out system errors, effective piezoelectric coefficient of periodically polled LiNbO_3_ (PPLN) was measured by the same PFM technique as standard reference (supplementary information section 9). The results show typical periodic piezoelectric domain structure of the PPLN and an average d_33_ value (~7.6 pm/V) close to the expected value (~7.5 pm/V) provided by the PPLN manufacturer^21^, confirming the reliability and accuracy of the PFM technique in our experiment. Therefore the measured piezoelectric coefficient for 001NB here should be close to its true value.

It is interesting to observe that the measured piezoelectric coefficient is a function of the frequency (shown in Fig. 3d,e). In the range of 5–35 kHz, the higher the frequency applied, the lower is the piezoelectric coefficient d_33_. The dip at 35 kHz is likely to be caused by the following reasons: 1) It has been reported^19^,^22^,^23^ that for ferroelectric material systems, the piezoelectric coefficient d_33_ decreases as the frequency increases. The interface pinning^23^ of spontaneous polarization, resulting from surface charge due to the high surface-to-volume ratio of the nanobelt^19^, can cause the piezoelectric coefficient to decrease when the frequency increases. This explains the decrease of piezoelectric coefficient of 001NB as the frequency increases from 5–35 kHz. 2) The resonance frequency of our 001NB on Au/Ti/Si substrate for PFM characterization may be close to ~45 kHz, which is similar to the resonance frequency that has been reported previously for other single-crystal PMN-PT systems^24^. Therefore when the applied frequency is 45 kHz, 001NB is resonating with an enlarged vibration amplitude, such that a larger deformation of 001NB is detected by PFM. This makes the measured electromechanical response of 001NB to be stronger. This reason explains the increase of piezoelectric coefficient of 001NB as the frequency increases from 35 to 45 kHz. 3) The resonance frequency of the cantilever for PFM characterization is ~50 kHz. Therefore when the applied frequency is 45 kHz, the cantilever was resonating with a noticeable vibration amplitude, which results to a larger final signal generated by the lock-in amplifier in PFM. This reason also contributes to the increase of the measured piezoelectric coefficient as the frequency changes from 35 to 45 kHz. 4) Similar to the nanobelt reported previously^19^ for PFM characterization, the electrical contact between the bottom of 001NB in our project and the conductive layer on Si substrate is not perfect^19^, therefore the electrical contact condition changes when frequency increases. The measured electromechanical response of the 001NB is thus influenced as the frequency changes, contributing to the observed frequency dependence of d_33_. In sum, the four reasons mentioned above could function together to qualitatively explain the appearance of the dip at 35 kHz. To quantitatively and unambiguously explain this phenomenon in details, new experiments need to be carefully designed and performed.

**Figure 4:**
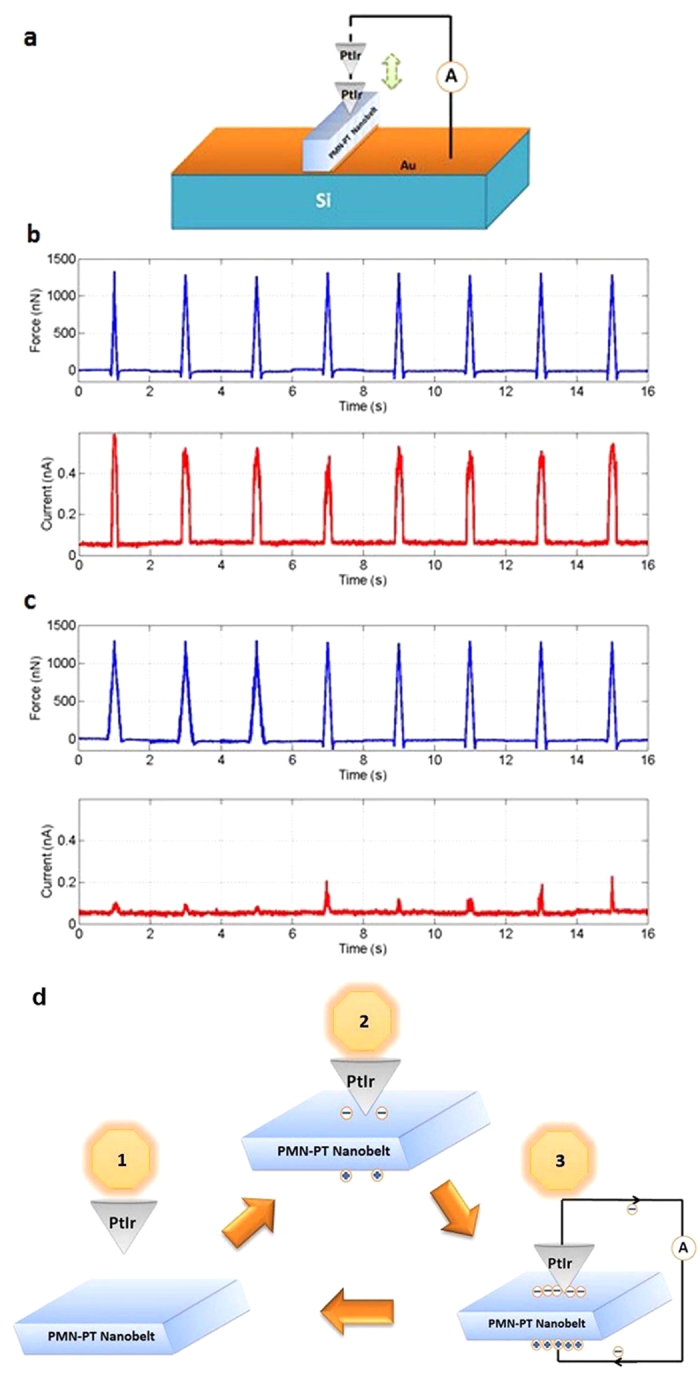
Sensing ability of a prototype nano-force sensor based on a single PMN-PT nanobelt (001NB). (**a**) The schematic illustration of the nano-force sensor. For testing purpose, the force exertion and current measurement were realized by the conductive AFM tip. (**b**) The periodic force application on 001NB and the periodic electrical current measured as a response of the force signal. (**c**) The same periodic force was applied on Au/Ti coated Si substrate and almost no electrical current was measured as a response of the force signal. (**d**) Schematic illustration of the current generation mechanism for the prototype nano-force sensor during deformation.

The stress sensing ability of the 001NB was demonstrated by a model system consisted of a Pt/Ir coated AFM tip and the 001NB on an Au/Ti coated Si wafer. By using the AFM tip as the stress generator (Fig. 4a), the mechanoelectrical transduction of the 001NB was measured as shown in Fig. 4b. The electrical current peak was generated because electrons would drift through the external circuit to balance the potential difference induced by the external pressure on the 001NB. To confirm that the current peak was truly induced by piezoelectric effect of the 001NB, a contrast experiment was performed on the Au/Ti coated Si substrate. This time almost no electric current was induced by the same amount of tip force (as shown in Fig. 4c). Therefore 001NB was proved to be a well-functioning nanoscale sensor/generator, potentially applicable in self-powered nanodevices. The current generation mechanism of 001NB is schematically illustrated in Fig. 4d. When AFM tip is above the sample, no stress is applied to the nanoscale sensor so that 001NB is in complete relaxed status and there is no piezopotential drop along [001]_c_ direction. Sequentially, when a mechanical pressure is applied onto the top and bottom surfaces of 001NB, a piezopotential is induced between the top and bottom surfaces. Electrons will drift through the external circuit to balance the potential difference, thus a current peak is observed. The reverse electrical current can not be detected because the external circuit is disconnected as the AFM tip is raised up.

**Figure 5:**
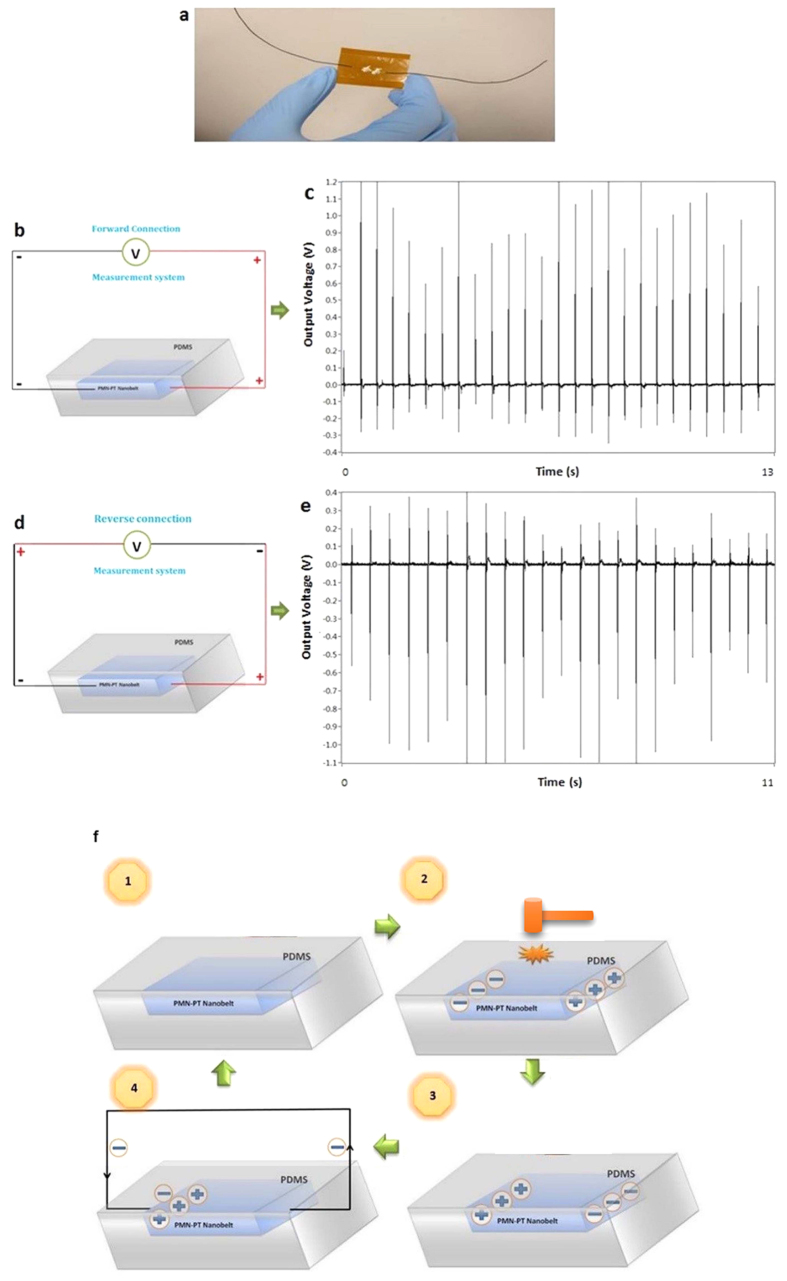
Performance of a lateral PNG based on a single PMN-PT nanobelt. (**a**) The assembled device before being capped with a thin layer of polydimethylsiloxane (PDMS) for protection. (**b**) The schematic illustration of the forward-connection set up. (**c**) A typical voltage generation graph of the single PMN–PT nanobelt-based lateral PNG when forwardly connected. (**d**) The schematic illustration of the reverse-connection set up. (**e**) A typical voltage generation graph of the PMN–PT nanobelt-based lateral PNG when reversely connected. (**f**) Schematic illustration of the voltage output mechanism for the lateral PNG based on a single PMN-PT nanobelt.

The energy scavenging ability of the FIB-cut PMN-PT nanobelt was further demonstrated by a lateral PNG built on it, as shown in Fig. 5a. The orientation of the single nanobelt during device assembling was well controlled so that signal was generated along [001]_c_ direction (the bulk PMN-PT was poled along [001]_c_ direction before FIB-cutting to ensure maximum performance, see supplementary information section 10). To protect the device from cyclical mechanical deformation during testing, it was capped with a thin layer of polydimethylsiloxane (PDMS). For testing purpose, a small hammer was used to tap the PNG for voltage generation, representing its actual application for energy harvesting/force sensing. When the PNG was forward-connected (Fig. 5b), a typical voltage generation graph was recorded as in Fig. 5c. Positive voltage peaks ranging from ~0.6 to ~1.2 V were repeatedly generated in an open circuit under mechanical tappings. Upon each direct impact, a large positive peak was generated, followed by a negative peak corresponding to the damping effect resulting from the removal of the initial stress and the relaxation of device. The negative voltage peaks ranged from ~ −0.1 to ~ −0.4 V. The intensity difference between the positive and negative signals was from the capacitor nature of the nanobelt-based device. The charges induced along with the positive voltage peak would be stored on the surface of the electrodes. When an opposite voltage signal was generated, the stored charges would first be consumed. Consequently the measured negative voltage would always be much smaller than the positive voltage signal. To confirm that the output signals were truly generated by the piezoelectric effect of the PMN–PT nanobelt, a switching-polarity test (i.e. reverse connection) was performed, as shown in Fig. 5d. This time a negative peak was generated first, followed by a smaller positive signal afterwards. The generated positive voltage peaks ranged from ~0.1 to ~0.4 V, while the negative voltages were in the range of ~ −0.5 to ~ −1.2 V, which can be observed in Fig. 5e. The output voltage can be expressed as a product of length, piezoelectric voltage constant and internal stress of the PMN-PT nanobelt:


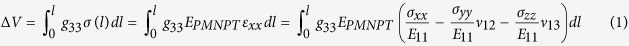


where l is the length of the nanobelt across two electrodes, and g_33_ is the piezoelectric voltage constant (38.8 × 10^−3^ V m/N)^25^. ε_xx_ and σ(l) are the lateral strain and stress within the PMN-PT nanobelt. σ_xx_, σ_yy_, and σ_zz_ are the stresses of x (length), y (width) and z (height) directions. ν_12(3)_ and E_11_ are the Poisson’s ratio and modulus of the whole system composed of the PMN-PT nanobelt, the flexible polyimide (PI) substrate and the thin layer of polydimethylsiloxane (PDMS):


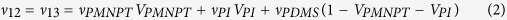



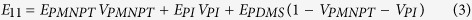


For the largest absolute value of voltage output (~1.2 V), the corresponding maximum lateral strain of the PMN-PT nanobelt was obtained as:


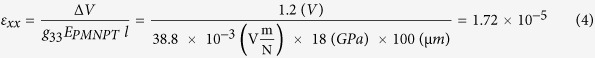


Therefore the output voltage of the PNG resulted from the large piezoelectric response of PMN-PT and the design of structures, instead of huge external impact. The small strain within the PMN-PT nanobelt is beneficial for its mechanical integrity/stability during operation and its cyclic life for application, since it effectively reduces the risk of potential fracture or damage of the piezoelectric materials under high-frequency vibrational conditions, broadening their safety vibration frequency and amplitude range. It also demonstrates its high sensitivity towards small-level mechanical disturbances. The voltage output mechanism of the lateral PNG based on a single PMN-PT nanobelt is schematically illustrated in Fig. 5f. At first, no stress is applied to the PNG so that the PMN-PT nanobelt is in complete relaxed status. Sequentially, when a mechanical pressure is applied onto the top and bottom surfaces of the PNG, a piezopotential is induced along the lateral direction of the nanobelt due to the poisson’s effect. Electrons will drift from the negatively charged (V−) side to the positively charged (V+) side through the external circuit, leaving the V− region electron-depleted. When the loading pressure on the nanobelt is gradually released and the lateral strain decreases from its maximum value to zero, the previously V− side turns to be the positive side and a transient negative voltage output peak will be observed. The accumulated electrons flow back through the external circuit to balance the reversed potential difference, and the output voltage will eventually turn into zero. The nanobelt as well as the whole PNG will also turn back to zero-strain status.

## Conclusions

In summary, a top-down method has been adopted to successfully fabricate single-crystal PMN-PT nanobelt with precisely-controlled crystal orientation and dimensions. The electromechanical coupling of the PMN-PT nanobelt was carefully studied to rule out system errors. The measured piezoelectric constant of the PMN-PT nanobelt is the highest to date, outperforming the previously reported PMN-PT nanowires by 50 percent. It is also about 4 and 21 times higher than the maximum reported values of one dimensional PZT and ZnO nanostructures. The top-down method here thus provides a handy way to prepare individual nanostructures with desirable dimensions and orientations. Furthermore, the control over the crystal orientation of the PMN-PT nanobelt during device assembling permits us to exploit the best performance out of the material. Lateral PNG based on a single PMN-PT nanobelt is realized, with an output voltage ~4 times higher than that of a single-CdTe PNG^12^, ~13 times higher than that of a single-ZnSnO_3_ PNG^11^, and ~26 times higher than that of a single-ZnO PNG^9^,^10^. The present research demonstrates a new method to prepare a single-crystal PMN-PT nanostructure with high piezoelectric performance that is readily available for energy harvesting. The profoundly increased output voltage of the lateral PNG based on a single PMN-PT nanobelt shows the potential application of PMN-PT nanobelt for energy scavenging and self-powered devices, thus enriching the material choices of this field.

## Methods

### Sample preparation

Bulk PMN-PT substrate (5 × 5 × 0.5 mm) is purchased from MTI Corporation. The out-of-plane orientations of the top/bottom surface and side planes of the as-received PMN-PT substrate are <100>_c_. The Strata DB-235 dual-beam (FIB/SEM) system was used to cut nanobelts and nanorods from the PMN-PT bulk. An omni-probe was used to transfer and manipulate the FIB-cut nanostructures after cutting, for piezoelectric characterization and device assembling.

### Structural and compositional characterization

A Bruker D8 Discover X-Ray Diffractometer was used to perform XRD θ–2 θ scan. Leica DCM 3D Micro-optical System was used for confocal and interferometry characterization. FEI Quanta 200 FEG Environmental-SEM was used for SEM imaging and EDX analysis. TEM and high-resolution TEM observations were performed for the PMN-PT nanobelt on a Philips CM200 FEG-TEM operated at 200 kV.

### PFM characterization of the 001NB

The FIB-cut 001NB was transferred to a silicon substrate covered by 10 nm of Ti and 100 nm of Au for conduction purpose. The out-of-plane orientation is maintained during the transfer process, enabled by an omni-probe. 5 nm of Pt was coated by FIB on (001)_c_ surface of the nanobelt, serving as an electrode to ensure a uniform electric field and avoid electrostatic effects^19^. The simultaneous AFM and PFM characterizations were performed using a Veeco Nanoman AFM. Conductive AFM tip (NSG-10) was used to apply voltage on the nanobelt.

To measure the piezoelectric coefficient of 001NB, it was firstly located by contact mode AFM, and then the AFM tip was landed right onto the center of the nanobelt by point-and-shoot function. Sequentially the ramp-plot function was used to ramp the applied voltage from −10 to 10 V, generating a piezoelectric displacement vs. voltage sweeping curve. Since the displacement direction under the applied drive voltage was parallel to the electric field, the coefficient obtained was effective d_33_. A total of 10 piezoelectric displacement vs. voltage curves were measured at two different positions on 001NB, by systematically changing the frequency of the applied AC signal from 5 kHz to 45 kHz at each position. All the other measuring parameters were maintained to be the same.

### Device assembling

After poling of the PMN-PT bulk, a nanobelt was cut in the way such that its long side (100 μm) was along the poling direction. The nanobelt was sequentially transferred onto a flexible polyimide (PI) substrate (4 cm length, 3 cm width, and 0.01 cm thickness). Silver paste was applied carefully to both ends of the nanobelt for fixing and electrical conduction purposes, and two conductive wires were bonded to each end for electrical measurement.

## Additional Information

**How to cite this article**: Wu, F. *et al.* Energy scavenging based on a single-crystal PMN-PT nanobelt. *Sci. Rep.* **6**, 22513; doi: 10.1038/srep22513 (2016).

## Supplementary Material

Supplementary Information

## Figures and Tables

**Table 1:** Effective piezoelectric coefficients d_33_ measured from different positions on (001)_c_ surface-dominated PMN-PT nanobelt, with various frequencies of the AC signal applied on the sample.

Data point	Slope	Std error	d_33_(pm V^−1^)
Position 1, f = 5 kHZ	0.5519	0.0022	551.9
Position 1, f = 15 kHZ	0.4911	0.0019	491.1
Position 1, f = 25 kHZ	0.4370	0.0013	437
Position 1, f = 35 kHZ	0.3967	0.0009	396.7
Position 1, f = 45 kHZ	0.4596	0.0015	459.6
Position 2, f = 5 kHZ	0.4711	0.0016	471.1
Position 2, f = 15 kHZ	0.4374	0.0012	437.4
Position 2, f = 25 kHZ	0.4433	0.0014	443.3
Position 2, f = 35 kHZ	0.4130	0.0010	413
Position 2, f = 45 kHZ	0.3828	0.0008	382.8
**Average**	**0.4484**	**0.0014**	**448.4**
