# Supplementary material for: Energy scavenging based on a single-crystal PMN-PT nanobelt
Source: Sci Rep. 2016 Mar 1;6:22513. doi: 10.1038/srep22513 (PMC4772540; doi:10.1038/srep22513)
Supplement: Supplementary Information [file srep22513-s1.pdf]

Supplementary information

Energy scavenging based on a single-crystal PMN-PT nanobelt

Fan Wu, Wei Cai, Yao-wen Yeh, Shiyou Xu and Nan Yao

Princeton Institute for the Science and Technology of Materials (PRISM), Princeton University, 70 Prospect Avenue, Princeton, New Jersey 08540, USA

Section 1: Importance of piezoelectric nanostructures

Section 2: Importance of PMN-PT nanostructures

Section 3: Effect of composition, crystal structure and orientations on the piezoelectric properties of PMN-PT nanostructures

Section 3.1: Effect of compositions and crystal structures

Section 3.2: Effect of crystal orientations

Section 4: Advantages of using FIB to prepare PMN-PT nanostructures with controlled shape, size and orientations in a repeatable manner

Section 5: 3D confocal microscopic characterization of FIB-cut nanostructures

Section 6: SEM characterization of FIB-cut nanostructures

Section 7: Piezoresponse Force Microscopy (PFM) principles in this project

Section 8: AFM height map and PFM phase map of 001NB

Section 9: PFM characterizations of periodically poled lithium niobate (PPLN) as reference

Section 10: Poling of the bulk PMN-PT substrate along $[001]_c$ direction

Section 11: Detailed mathematical model for voltage generation of the lateral PNG built on a single PMN-PT nanobelt

Section 12: Supplementary References

Section 1: Importance of piezoelectric nanostructures

Piezoelectric nanostructures have attracted extensive attention because they can convert mechanical deformation directly into electrical charges, thus scavenge mechanical energy from the environment to drive nano-devices/systems, or enable novel self-powered sensing devices [1]. Piezoelectric and ferroelectric materials, such as lead zirconate titanate (PZT) [2], (1−x) $Pb(Mg_{1/3}Nb_{2/3})O_3$− x $PbTiO_3$ (PMN-PT)[3], $BaTiO_3$ [4], ZnO [5], poly(vinylidene fluoride)(PVDF) [6], etc., have been intensively studied as effective and efficient building blocks for devices converting ambient mechanical energy into electricity [4,7,8]. Based on these materials, a variety of micro- or nanoelectromechanical systems (MEMS or NEMS) were developed for harvesting energies from random vibrations, mechanical waves, or body movements like walking, running, or typing [9-11]. For example, piezoelectric ZnO nanowires (NWs) have been demonstrated to successfully harvest micro- and nanoscale mechanical energy [12,13]. Piezoelectric nano-structures/devices have wide applications in fields requiring accurate displacement, quick response time, high generative force, or small device size[14].

Generally for a piezoelectric nanodevice, the strain energy density (which is a measure of the energy per unit mass) is the most important parameter among all the material properties. The strain energy density E_{max} can be calculated as:

$$E_{max} = \frac{1}{4\rho}\frac{Y\,\varepsilon_{max}^2}{2} \qquad (1)$$

where Y is the elastic modulus of the device, ε_{max} is the maximum field induced strain, and ρ is the device's density[14]. Density and elastic modulus change little from material to material for piezoelectric materials, therefore the level of strain and maximum strain achievable with a reasonable electric field dominate the energy density. For a given electric field, the piezoelectric coefficient (d_{ij}) determines the induced strain level, thus is the most widely pursued parameter for high-performance piezoelectric devices.

Section 2: Importance of PMN-PT nanostructures

The piezoelectric coefficient (d_{33}), which represents the ability of piezoelectric materials to convert mechanical deformation into an electric signal, plays a key role in the device performance. $BaTiO_3$ and $NaNbO_3$ based piezoelectric nanocomposites have limited output voltage and current due to their relatively low piezoelectric coefficients. Materials with high piezoelectric constants are highly desired for applications of piezoelectric materials. One such material is the lead-based relaxor ferroelectric (1−x) $Pb(Mg_{1/3}Nb_{2/3})O_3$− x $PbTiO_3$ (PMN-PT), which exhibits strain levels and piezoelectric coefficients 5 to 10 times higher than bulk PZT ceramics and a large electromechanical coupling coefficient of $k_{33} \sim 0.9$ [15,16]. PMN-PT bulk was reported to have a d_{33} up to 2500 pm/V [17], which was almost 30 times higher than that of $BaTiO_3$ (approximately 85.3 pm/V [18]) and 4 times higher than that of PZT bulk material. For PMN–PT nanowires, d_{33} was measured as 371 pm/V [3], which was over 13 times higher than that of $BaTiO_3$ nanoparticles (28 pm/V [19]) and 90 times higher than that of $NaNbO_3$ nanowires (4 pm/V [20]), respectively. A theoretical prediction [8] has suggested that PMN–PT nanowires could generate high output power with higher efficiency than other piezoelectric nanostructures. This is because the ultrahigh dielectric constants lead to a large intrinsic capacitance, such that only a small external resistant load is needed to extract the power out.

Furthermore, nanostructures can undergo large deformation [21,22] due to their excellent mechanical properties (e.g. the maximum strain for ZnO Nanowire is ~7.7%[23], while the maximum strain for bulk ZnO material is ~0.2%[24]), thus producing larger electric charges and higher output power, rendering them attractive for energy applications. The high flexibility and strain tolerance of nanostructures can effectively reduce the risk of potential fracture or damage of piezoelectric materials under high-frequency vibration conditions, thus broadening their safety vibration frequency and amplitude range [25]. As electromechanical systems move to nanoscale, the dense integration of nanoscale devices to provide novel system functionality is highly desired. The mechanical systems are required to be controlled by low voltage signals provided by small, cheap, densely integrated analog or digital circuits[26]. Owing to the small size and high flexibility of nanostructures, the nano-devices based on them are very sensitive to small-level

mechanical disturbances and are ideal for powering wireless sensors, microrobots, NEMS/MEMS, and bioimplantable devices[11,27].

Therefore, PMN-PT nanostructures are of great potential being used as the fundamental building block for higher power nanogenerators, high sensitivity nanosensors, and large strain nanoactuators. A *device based on PMN–PT nanostructures would have great potential for high-power nanogenerators and large-output signal sensors with lightweight and great flexibility.*

Section 3: Effect of composition, crystal structure and orientations on the piezoelectric properties of PMN-PT nanostructures

Section 3.1: Effect of compositions and crystal structures

The piezoelectric properties of PMN-PT nanostructures depend on both intrinsic (stoichiometry, orientation) and processing-related (phase purity, defect density) factors. (1-x)PMN-xPT crystal is a complex solid solution of relaxor ferroelectric PMN and normal ferroelectric PT. The structure of (1-x)PMN-xPT crystal transforms from rhombohedral to tetragonal phase, depending on the composition x at room temperature. When PT concentration (x) is below 30%, PMN-PT has a rhombohedral (3m) structure, while at PT concentrations larger than 35%, the crystal exhibits the tetragonal (4mm) symmetry. When PT content is located within the range of 0.30-0.38, which is the so-called morphotropic phase boundary (MPB) region, a monoclinic phase exists [28]. PMN-PT exhibits its strongest piezoelectric effects when the composition is situated near MPB between two distinct crystalline structures [29].

Apart from composition, another important parameter influencing the piezoelectric properties of PMN-PT nanostructures is crystal orientation, which will be described in section 3.2.

Section 3.2: Effect of crystal orientations

<111> polarized PMNPT:

Since the spontaneous polarization in each unit cell of PMN-PT is along one of the eight <111> directions of cubic coordinates[30], complete poling along <111> direction results in a single domain state, which is unstable, leading to domain reorientation and depoling. Complete domain orientation and single domain configuration under bias may cause elastic energy in crystals to increase, however, resulting in depoling after removing electrical field. The amount of hysteresis can be translated into degree of depoling, because more depoling involves more domain reorientation, which results in increased hysteresis. The large remnant strain values (after removal of electrical field) and domain instability result to the inferior piezoelectric properties of <111> oriented rhombohedral PMN-PT crystals. The electromechanical coupling coefficient k_{33} and the piezoelectric constant d_{33} for single-domain (poled along [111] direction) 0.67PMN–0.33PT are 69% and 190 pC/N, respectively [31].

<100> polarized PMNPT:

In contrast, the domain configuration of <001> polarized rhombohedral crystals was found to be stable [14]. At room temperature, rhombohedral PMN-PT phase with 3m symmetry has eight possible dipole orientations along the body diagonal directions (< 111> family). When an electric poling field is applied to the crystals along <001> of the cubic axes, a multi-domain configuration can be produced consisting of four degenerate states and charged domain walls, i.e. <001> poled crystals have the configuration that each domain has one of four possible polar directions <111>, <-111>, <1-11>, and <-1-11>. Therefore remnant polarization of <001> oriented rhombohedral crystal will be $1/\sqrt{3}$ of <111> oriented crystal's [14]. <001> poled PMN-PT crystals will exhibit almost hysteresis-free strain behavior and ultrahigh piezoelectric coefficient (d_{33}) as a consequence of domain stability. Multi-domain 0.67PMN- 0.33PT single crystals show extraordinarily large k_{33} of 94%, and d_{33} of 2500 pC/N at room temperature [31].

<110> polarized PMNPT:

The values of d_{33} along the <001> and <011> directions are nearly equivalent over the temperature range of 25-80 °C [32].

Section 4: Advantages of using FIB to prepare PMN-PT nanostructures with controlled shape, size and orientations in a repeatable manner

Since the piezoelectric properties of PMN-PT nanostructures are extremely sensitive to the parameters mentioned above, *it is of critical importance to create PMN-PT nanostructures with controlled composition, crystal structure and orientations in a repeatable manner.*

Previously, 1-D PMN-PT nanostructure was first synthesized through a bottom-up approach[3]. The hydrothermally synthesized PMN-PT nanowires showed a piezoelectric constant up to 381 pm/V, with an average value of 373 ± 5 pm/V [3]. This value is about 15 times higher than the maximum reported value of 1-D ZnO nanostructures [33] and 3 times higher than the largest reported value of 1-D PZT nanostructures [34]. The hydrothermally synthesized PMN-PT nanowires [3] were then used as the core building block for a flexible piezoelectric device [1], which demonstrated significantly improved performances for energy harvesting and self-powered sensing. The ground-breaking PMN–PT nanowire-based device demonstrated an output voltage up to 7.8 V and an output current up to 2.29 μA (current density of 4.58 μA/cm^2) [1]. The output voltage is more than double that of other reported piezoelectric nanocomposites, and the output current is at least 6 times greater [1], which demonstrated the promising applications of PMN-PT nanostructures. However, the hydrothermal synthesis process of PMN-PT nanowires has the following drawbacks:

(1): Impurity elements were introduced during the hydrothermal synthesis process.

During hydrothermal synthesis, potassium reacts with niobium and produces $KNbO_3$ [3], which is also a well-known piezoelectric material, but with piezoelectric constants (d_{33}= 7.9 pm/V [35]) much smaller than that of PMN-PT. Consequently the piezoelectric properties of PMN-PT nanowires with potassium residue were degraded.

(2): Crystal structure may be distorted by impurity atoms.

Impurity (potassium) atoms diffused into the nanowire under high temperature and high pressure environment [3], thus the crystal structure may be distorted, though the major lattice remained unchanged. Since the piezoelectric effect originates from the special crystal structure of ferroelectric materials, the distortion of crystal structure by impurity atoms will degrade the piezoelectric properties too.

(3): Defect density may increase due to lattice distortion.

As impurity atoms occupy substitutional or interstitial positions in the lattice, they turn into point defects in the hydrothermally synthesized PMN-PT nanowires. Also linear defects (such as edge dislocations and screw dislocations) and planar defects (such as twins and stacking faults) will be introduced if the concentration of impurity atoms reachs a certain level.

(4): Out-of-plane orientation of PMN-PT nanowires can not be controlled.

As manifested by the XRD Θ-2Θ patterns in ref [3], X-ray peaks corresponding to (100), (111), (210), and (211) planes of perovskite PMN-PT existed for hydrothermally synthesized PMN-PT nanowires. This means different PMN-PT nanowires have different out-of-plane orientations, which are out of control for hydrothermal synthesis method. Since millions of nanowires were synthesized in the same batch and laid down on the substrate randomly, it is impractical to demand the manipulation of the out-of-plane orientations of PMN-PT nanowires.

(5): Precise control of shape and size of an individual nanowire is difficult.

Similarly as pointed out in point (4), the precise control of shape and size of an individual nanowire is also impossible for a bottom-up method, such as hydrothermal synthesis.

(6): PMN-PT composition may be influenced by excess chemical reaction products.

According to the quantitative analysis of hydrothermally synthesized PMN-PT nanowires [3], the ratio of PMN and PT in nanowires is about 1.82, lower than the designed composition of 1.86 (65/35). The ratio between magnesium and niobium (4.10/8.89) is lower than 1:2, with the loss of magnesium. Furthermore, the concentration of lead is less than that in the precursor.

However, all the drawbacks mentioned above can be overcome by FIB method used in this project. The as-received PMN-PT bulk substrate is grown by Bridgman method, with $[001]_c$ out-of-plane orientation ($\pm$0.5 Degree) and (010) side planes. The composition is 0.67PMN-0.33PT, which is within the MPB region and ensures the good piezoelectric properties. *Consequently, the composition, purity and crystal structure of PMN-PT are guaranteed for FIB cutting.* By tilting and rotating the sample relevant to the incident ion beam during FIB cutting, nanobelts with any desirable out-of-plane orientations (such as $<100>_c$, or $<011>_c$) and nanostructures with desirable shapes (such as nanorods/wires) can be obtained by FIB. *Therefore the size, shape, and out-of-plane orientation of FIB-cut nanostructures are precisely controlled.* The FIB cutting of a $(011)_c$ surface dominated nanobelt and a nanorod are shown in Figure S1 a and b, respectively. The characterizations of these FIB-cut nanostructures by 3D confocal microscopy and SEM will be demonstrated in the following sections. Furthermore, after being cut-free from the bulk PMN-PT, the single PMN-PT nano-belt/rod/wire can be picked up by the omni-probe and transferred to any substrate, such as the Au/Ti coated Si substrate and flexible polyimide (PI) substrate used in this project, for either piezoelectronic characterization or device fabrication. *Thus all the parameters (including out-of-plane orientation) of a single FIB-cut nanostructure can be maintained during the post-cut transferring processes, which is unfulfillable for hydrothermal synthesis method.* Last but not least, the side planes of the FIB-cut nanostructures were finished with low-current polishing (~50 pA), *such that the obtained nanostructures have a low density of defects or are even defect-free* [33].

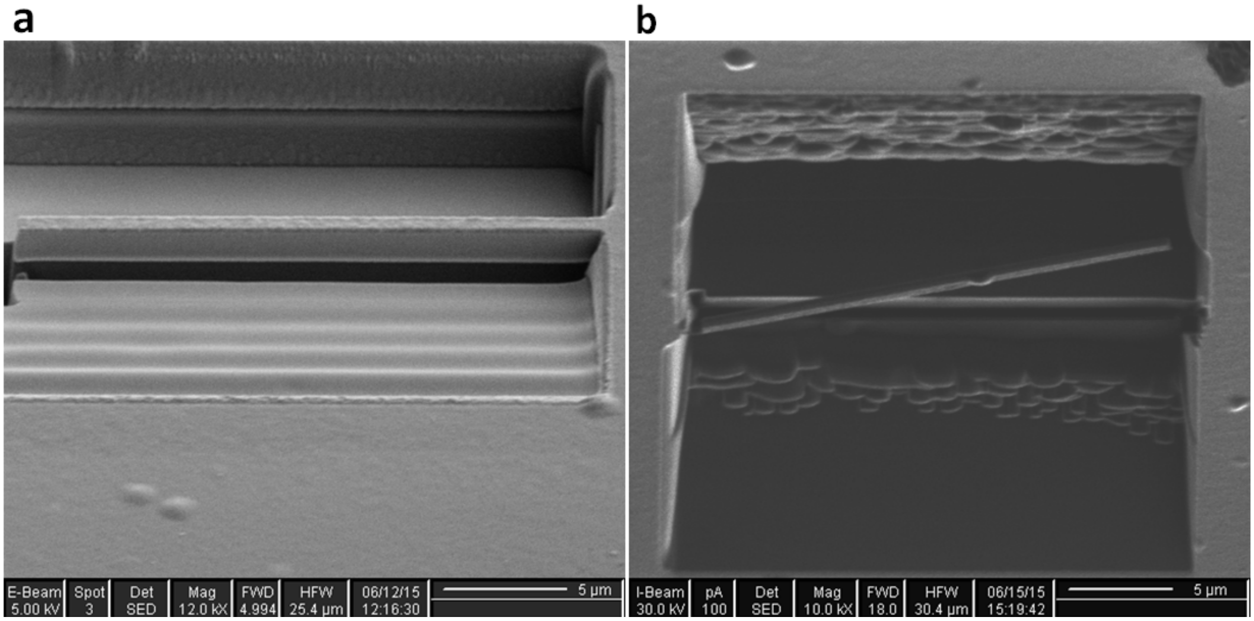

Figure S1. The FIB cutting images of different nanostructures. a, $(011)_c$ surface-dominated nanobelt; **b,** $(001)_c$ surface-dominated nanorod.

Section 5: 3D confocal microscopic characterization of FIB-cut nanostructures

After being cut free from the bulk, the $(011)_c$ surface dominated PMN-PT nanobelt was lifted out and observed by 3D confocal microscopy, combining confocal and interferometry technology for high-resolution non-invasive morphological inspection. The 3D topographical map (Figure S2a) of the $(011)_c$ surface dominated PMN-PT nanobelt shows its length and width to be ~22 μm and ~6 μm, respectively. The line profile (Figure S2b) derived from Figure S2a shows an average thickness of 300nm for the nanobelt.

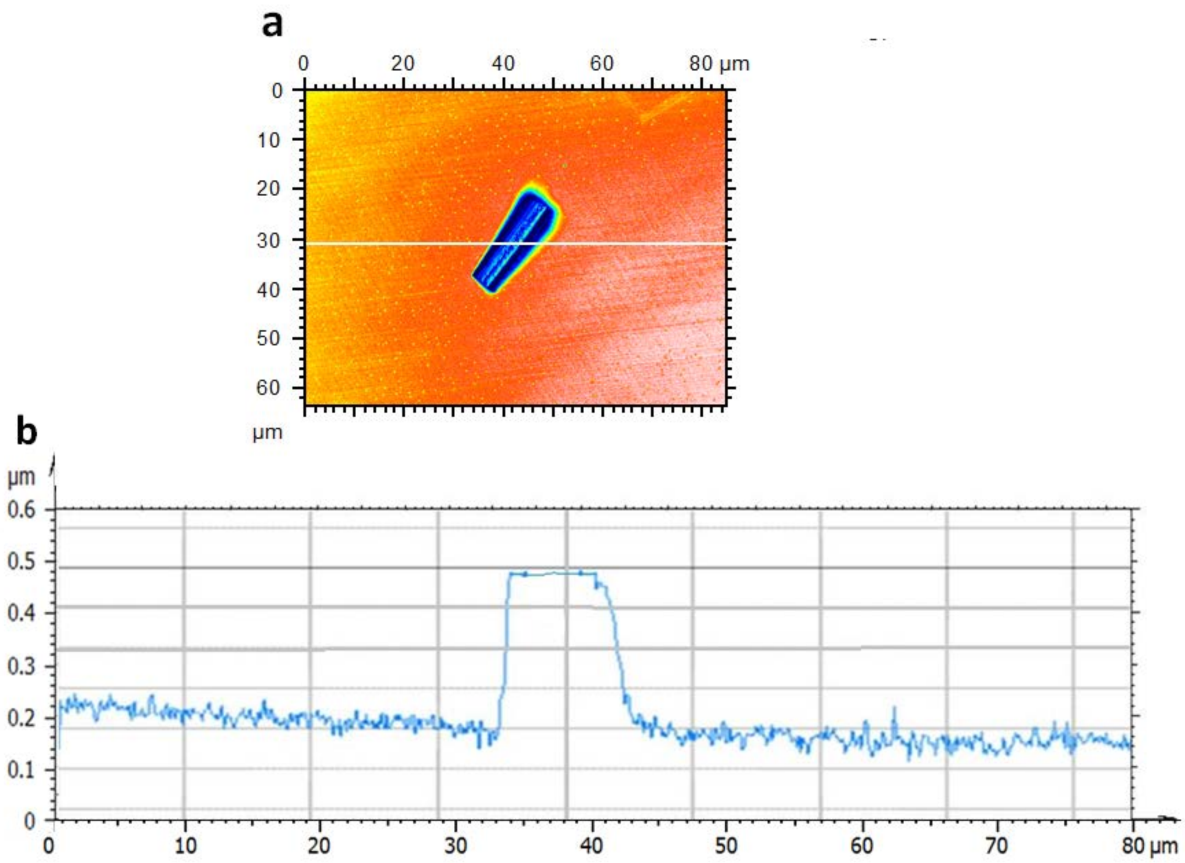

Figure S2. The topographical analyses of FIB-cut (011)$_c$-surface dominated nanobelt by confocal microscopy. a, 3D topographical map; **b,** line profile derived from panel **a**.

The 3D topographical map (Figure S3a) of the (001)$_c$ surface dominated PMN-PT nanorod shows its length and width to be ~24 µm and ~1 µm, respectively. The line profile (Figure S3b) derived from Figure S3a shows an average thickness of ~350nm for the nanorod.

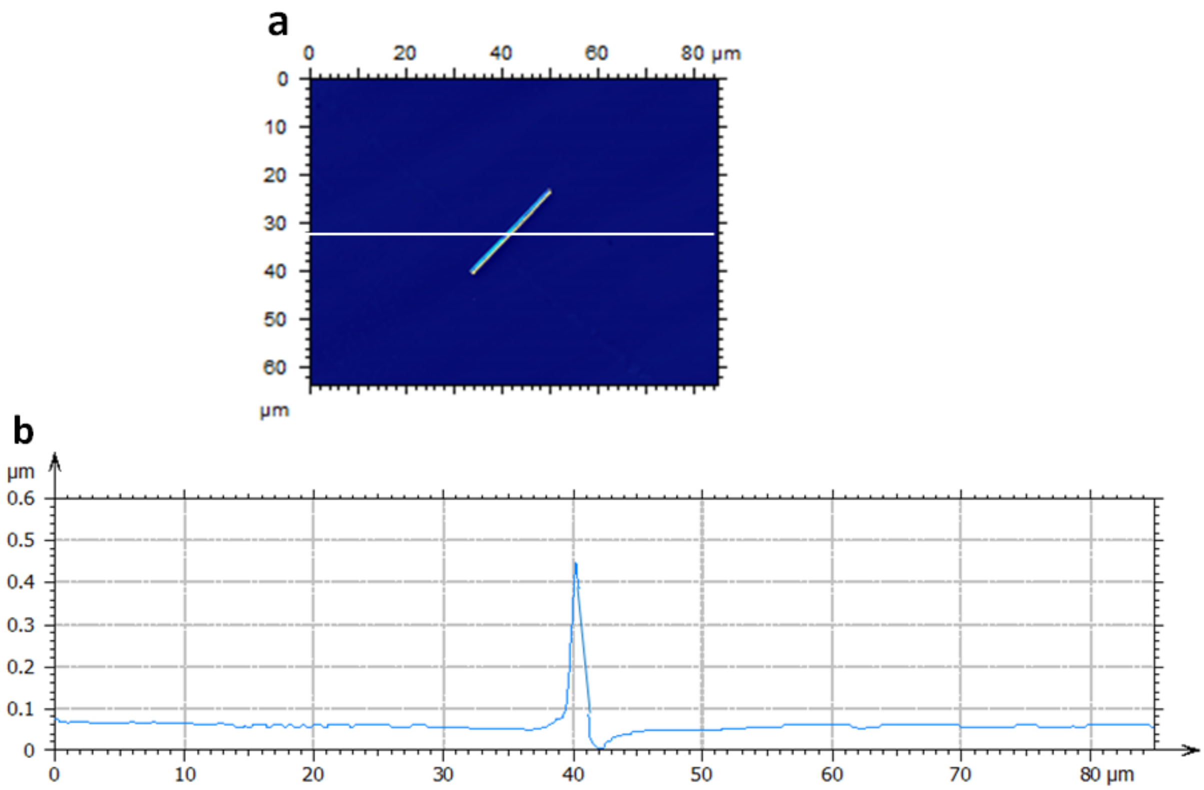

Figure S3. The topographical analyses of FIB-cut $(001)_c$-surface dominated nanorod by confocal microscopy. a, 3D topographical map; **b,** line profile derived from panel **a.**

Section 6: SEM characterization of FIB-cut nanostructures

The FIB-cut nanostructures were further studied by SEM for microstructural and compositional analyses. Figure S4a shows the morphology of the $(011)_c$ surface dominated PMN-PT nanobelt, which has a well-chiseled rectangular shape with sharp contours. The length and width of the nanobelt are measured from the SEM image to be ~19.8 and ~4.6 μm, respectively, corresponding well to the confocal microscopy results. Combining the line profile (Figure S2b) with SEM observation, the width-to-height and length-to-width ratios are obtained as ~ 15:1 and ~4.3:1, respectively. The energy dispersive spectrum (EDS) generated from the pink box in Figure S4a is shown in Figure S4b, in which Pb, Mg, Nb, Ti and O peaks are labelled.

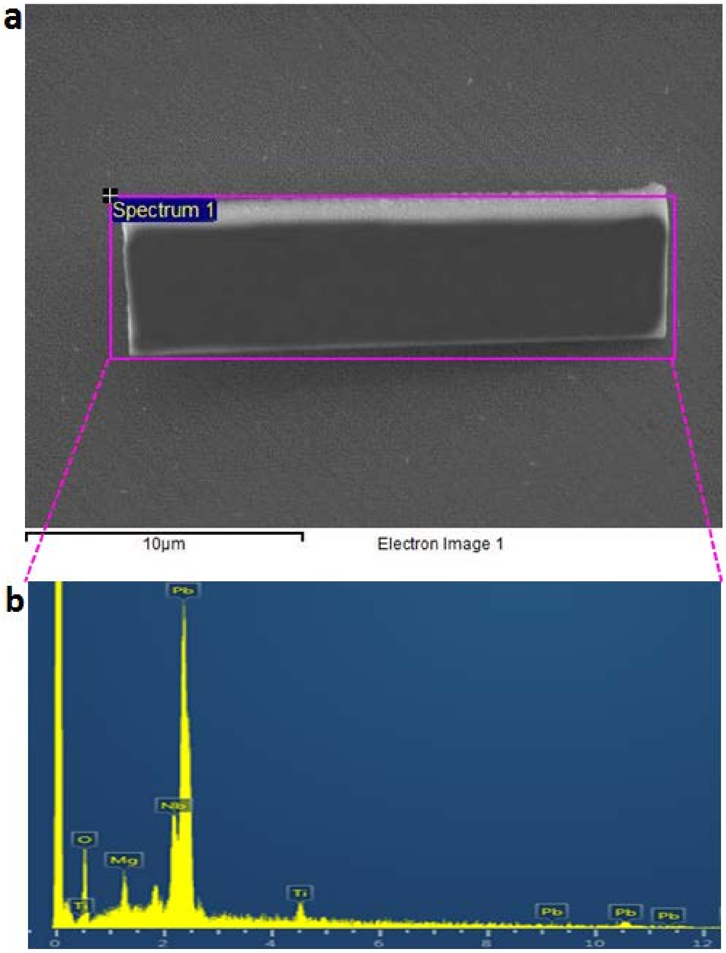

Figure S4. SEM characterization of the FIB-cut $(011)_c$ surface dominated PMN-PT nanobelt. a, SEM image showing the morphology with a well-defined rectangular shape with sharp contours. **b,** The energy dispersive spectrum (EDS) generated from the pink box in panel **a**.

Figure S5a shows the morphology of the $(001)_c$ surface dominated PMN-PT nanorod, which has a well-chiseled shape with sharp contours. The length and width of the nanorod are measured from the SEM image to be ~23.7 and ~1.4 μm, respectively, corresponding well to the confocal microscopy results. Combining the line profile (Figure S3b) with SEM observation, the width-to-height and length-to-width ratios are obtained as ~ 4:1 and ~17:1, respectively. The energy dispersive spectrum (EDS) generated from the pink box in Figure S4a is shown in Figure S4b, in which Pb, Mg, Nb, Ti and O peaks are labelled.

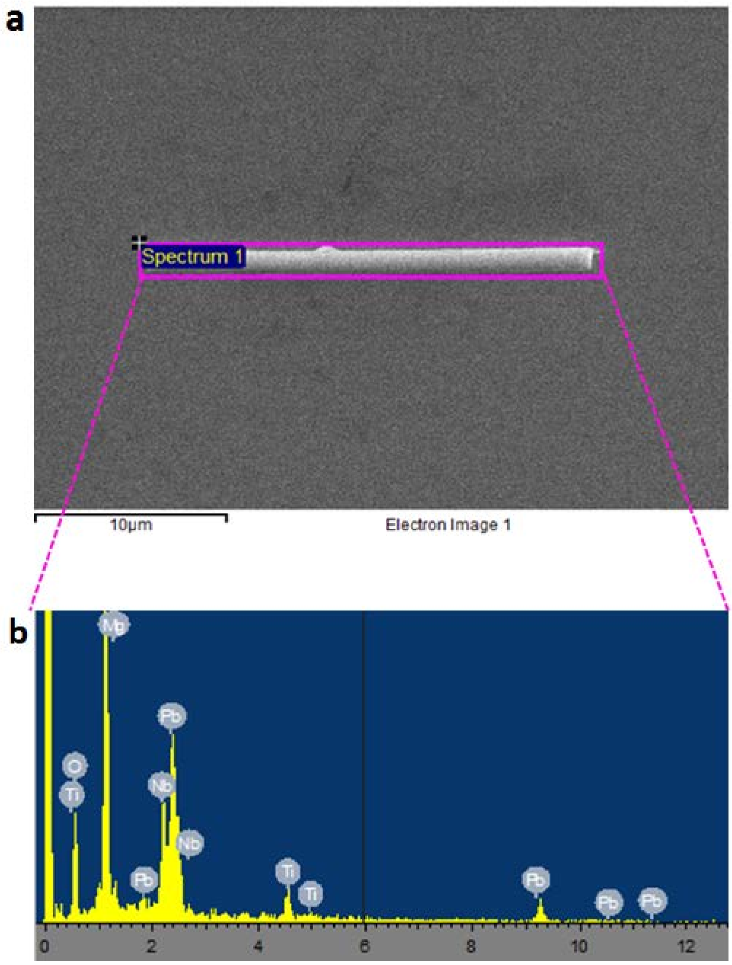

Figure S5. SEM characterization of the FIB-cut PMN-PT nanorod. a, SEM image showing the morphology of the FIB-cut PMNPT nanorod. **b,** The energy dispersive spectrum (EDS) generated from the pink box in panel **a.**

Section 7: Piezoresponse Force Microscopy (PFM) principles in this project

Piezoresponse force microscopy (PFM) is a powerful tool for probing local piezoelectric and ferroelectric properties of materials at nanoscale, first implemented by Güthner and Dransfeld [36]. The extremely small size of Atomic Force Microscopy (AFM) probe and the modern PFM techniques enable the characterization of piezoelectric properties on nanoscale with high resolution [37]. In our case, PFM was operated in vertical mode, in which the induced local vertical deformation was measured through the vertical deflection of the AFM cantilever and the out-of-plane polarization was studied. In a typical

PFM measurement, an AC driving voltage is applied to the specimen through the conductive cantilever tip, which induces a surface vibration of the specimen due to the converse piezoelectric effect (CPE), e.g:

$$\begin{bmatrix} X_1 \\ X_2 \\ X_3 \\ X_4 \\ X_5 \\ X_6 \end{bmatrix} = \begin{bmatrix} 0 & 0 & d_{31} \\ 0 & 0 & d_{31} \\ 0 & 0 & d_{33} \\ 0 & d_{15} & 0 \\ d_{15} & X_5 & 0 \\ 0 & 0 & 0 \end{bmatrix} \begin{bmatrix} E_1 \\ E_2 \\ E_3 \end{bmatrix} \tag{2}$$

where X_i is the strain tensor, d_{ki} is the piezoelectric tensor, and E_k is the electric field tensor. CPE describes how an applied electric field will create a strain, leading to a physical deformation of the material. In order to separate the low level signal from random noise, the deflection of the probe cantilever is detected by a standard photodiode detector and then demodulated using a lock-in amplifier. In our case, the reference oscillatory AC signal applied between the AFM tip and the Au surface during scanning is:

$$V_{\mathrm{ref}} = \mathrm{A}\cos(\omega t) \tag{3}$$

Where ω is the frequency of the reference signal, and the induced sample signal is:

$$V_{\mathrm{sample}} = \mathrm{B}\cos(\omega t + \varphi) \tag{4}$$

where φ is any phase shift between the two signals. Therefore the demodulator output (tip vibration signal) is obtained by multiplying the two signals together:

$$V_{tip} = \frac{1}{2}\mathrm{AB}\cos(\varphi) + \frac{1}{2}\mathrm{AB}\cos(2\omega t + \varphi) = V_{\mathrm{dc}} + V_{\mathrm{ac}}\cos(2\omega t + \varphi) \tag{5}$$

The AC component has a frequency of twice the original reference signal and the DC component is related to both the amplitude and phase of the input signal. The demodulator output is sent through a low-pass filter to remove the 2ω component and leave the DC component, and then the signal is integrated over a period of time. When the tip is scanned above the PMN-PT nanobelt, the responsive piezoelectric strain in the nanobelt will cause the displacement of the cantilever:

$$z = z_{\mathrm{dc}} + A(\omega, V_{\mathrm{ac}}, V_{dc})\cos(\omega t + \varphi) \tag{6}$$

Where φ is any phase shift between the reference and input signals. When the voltage is driven at a frequency (ω) well below the contact resonance of the cantilever, the above expression converts to:

$$z = d_{33}V_{\mathrm{dc}} + d_{33}V_{\mathrm{ac}}\cos(\omega t + \varphi) \quad (7)$$

Therefore the local piezoelectric response will be detected as the first harmonic component of the tip deflection, and phase φ of the electromechanical response generates information on the polarization direction below the tip. If the nanobelt expands vertically in response of a positive tip bias, the surface oscillations will be in phase with the tip voltage (φ = 0). If the response is opposite (nanobelt shrinks vertically), the surface oscillations will be out of phase with the tip voltage (φ = 180°). If the sample below the tip has no piezoelectric properties (e.g. Au layer in our case), there will be no phase relationship between the applied voltage and the surface oscillation. Generally, the magnitude of the oscillating response is a measure of the magnitude of d_{33} and the phase is sensitive to the polarization direction of the sample. The difference in φ is the reason of the contrast in the phase map (Figure S6).

When the ramp-plot function is used to ramp the applied voltage from -10 to 10 V and to generate a piezoelectric displacement vs voltage sweeping curve, the piezoelectric displacement (A_f in unit of nm), was obtained by multiplying the deflection signal (V_f in unit of mV) with the calibration constant (δ in nm/V) of the photodetector sensitivity, which is determined from the slope of the force–distance plot. We thus have:

$$A_f = \frac{\delta V_f}{16} \quad (8)$$

In which 16 is the gain factor used by the instrument. In our case, δ is measured to be ~50 nm/V.

Section 8: AFM height map and PFM phase map of 001NB

The simultaneously obtained AFM height map and PFM phase map of 001NB are shown in Figure S6a and b, respectively. The surface oscillations inside the nanobelt region is in phase with the tip voltage, which means φ = 0, (φ is the phase of the electromechanical response of the sample, see supplementary information section 7). Outside the nanobelt region, φ is random such that the contrast between the regions inside and outside of 001NB is obvious in phase map.

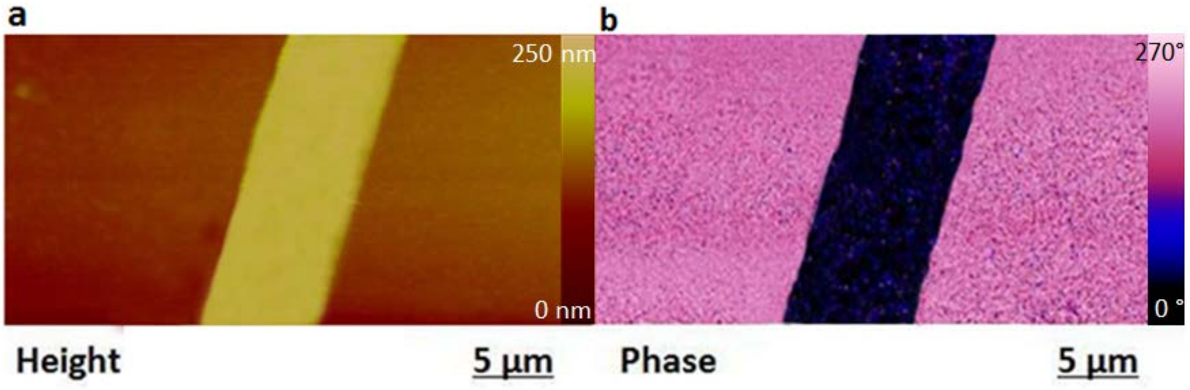

Figure S6. The simultaneous AFM and PFM characterizations of the $(001)_c$ PMN-PT nanobelt. a, AFM height map. **b,** PFM phase map.

Section 9: PFM characterizations of periodically poled lithium niobate (PPLN) as reference

To ensure the reliability and accuracy of the PFM measurement for PMN-PT nanobelt and rule out system errors, the effective piezoelectric coefficient of bulk $LiNbO_3$ was measured as standard reference by the same PFM technique. The height map, PFM phase map and amplitude map of periodically poled lithium niobate (PPLN) are shown in Figure S7. From the height map, the sample surface is observed to be flat (with a height variation range of ±5 nm), while the PFM phase and amplitude maps demonstrate the typical periodic piezoelectric domain structure of PPLN. The width of each piezoelectric domain is on the scale of ~5 µm.

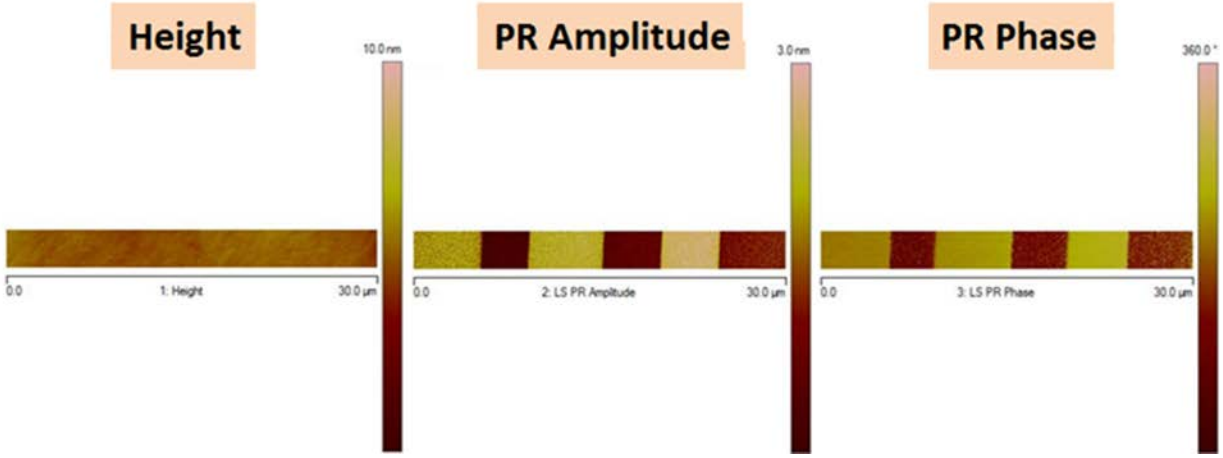

Figure S7. PFM characterization of periodically poled lithium niobate (PPLN) substrate as reference. The comparison among height, PFM amplitude and PFM phase maps demonstrates the typical periodic piezoelectric domain structure on the scale of ~5 μm.

To measure the piezoelectric coefficient of PPLN and compare it with the standard value, the piezoelectric displacement vs voltage curves from five different positions on PPLN are obtained and shown in Figure S8. From the slopes of the curves, the averaged effective piezoelectric coefficient for PPLN can be obtained as d_{33} = ~7.6 pm/V, which is close to the standard piezoelectric coefficient value (d_{33} = ~7.5 pm/V) provided by the PPLN manufacturer, confirming the reliability and accuracy of the measuring technique in our experiment. Therefore the measured piezoelectric coefficient for PMN-PT nanobelt should be close to its true value.

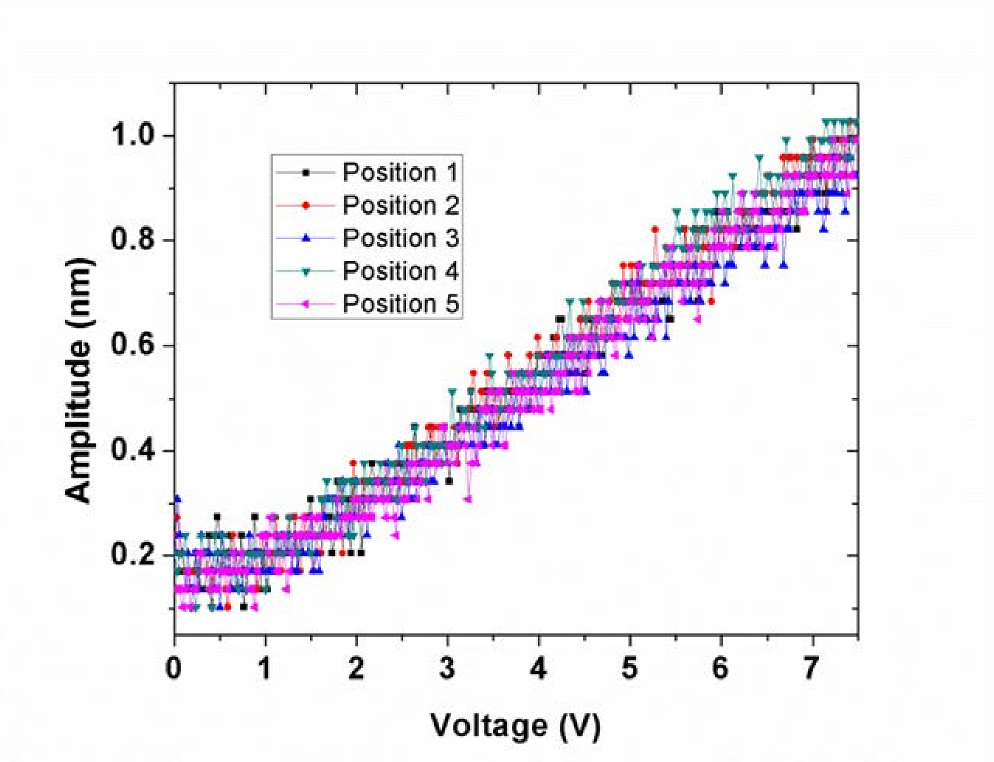

Figure S8. The piezoelectric displacement vs voltage curves from five different positions on periodically poled lithium niobate (PPLN).

Section 10: Poling of the bulk PMN-PT substrate along $[001]_c$ direction

To ensure good performances of the macroscopic sensor built on a single PMN-PT nanobelt, the bulk PMN-PT substrate (from which the nanobelt was FIB-cut) was poled along $[001]_c$ direction in a silicon oil bath at >100C for 24h before the FIB cutting. At room temperature, PMN-PT crystal with 3m symmetry has eight possible spontaneous dipole orientations along the body diagonal directions (<111> family), i.e. the spontaneous polarization in each unit cell of PMN-PT is along one of the eight <111> directions of cubic coordinates [30]. In a freshly made crystal, all eight directions exist in equal numbers. When an electric poling field is applied to the crystals along <001> of the cubic axes, a multi-domain configuration can be produced consisting of four degenerate states and charged domain walls, i.e. <001> poled crystals will have the configuration that each domain has one of four possible polar directions <111>, <-111>, <1-11>, and <-1-11>. These poling

directions are permanently realigned after poling, making the piezoelectric effect along <001>$_c$ direction much stronger. The poling setup and process for PMN-PT substrate before FIB-cutting are demonstrated in Figure S10 and can be divided into the following four steps:

First of all, the (001)$_c$ surface-dominated PMN-PT substrate (5mm in length, 5mm in width, 0.5mm in thickness) was put onto a SEM sample stub, which was electrically conductive and used as one electrode later during poling. A rubber ring (~15mm inner diameter, ~3mm in height) was then placed onto the SEM sample stub so that the PMN-PT substrate was in the center of the ring. The rubber ring placed here to isolate the two electrodes (the top sample stub and the bottom sample stub) because it is insulating.

Sequentially, another sample stub was put onto the rubber ring, working as the other electrode during poling.

Then the two electrodes (the top stub and the bottom stub) were connected to a high-voltage power supplier, so that the (001)$_c$ PMN-PT substrate was in a uniform electric field of ~5kV/cm. The whole system, include the inside region between two electrodes, was immersed in silicon oil to avoid electric breakdown in air.

Last but not least, the hot plate under the poling system was heated up to above 100 °C during the poling. The high temperature will assist the realignment of dipoles within each unit cell of the PMN-PT crystal. The poling was maintained in this configuration for 24 hours.

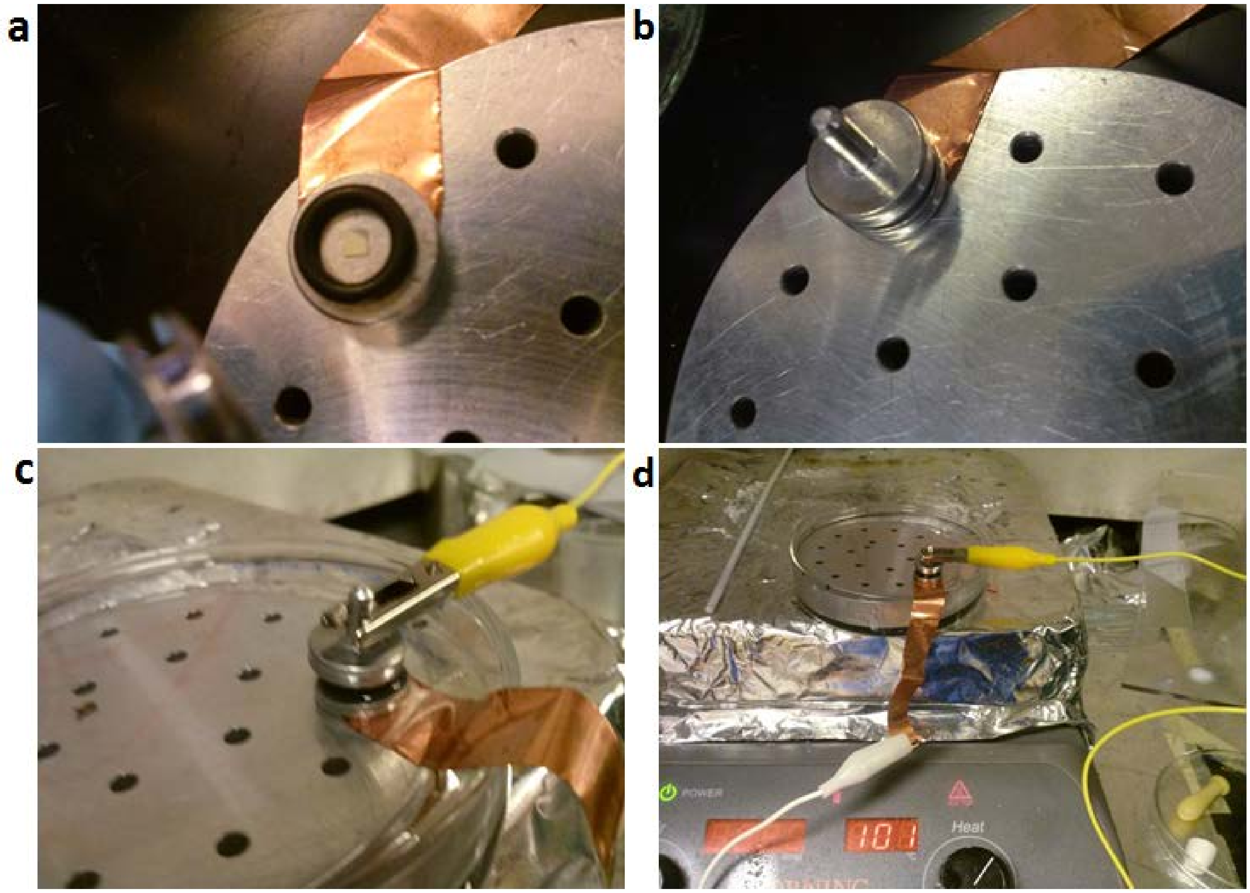

Figure S10. The poling setup and process for PMN-PT substrate before FIB-cutting. a, Put the PMN-PT substrate ($(001)_c$ as top surface) onto a sample stub, around which a rubber ring is placed. The rubber ring is used to isolate the two electrodes (the top stub and the bottom stub). **b,** Put another sample stub onto the rubber ring. **c,** Connect the two electrodes (the top stub and the bottom stub) to high-voltage power supplier, so that the $(001)_c$ PMN-PT substrate is in a uniform electric field of ~5kV/cm. The whole system is immersed in silicon oil to avoid electric breakdown in air. **d,** Heat up the whole system to above 100 °C during the poling. The poling lasts for 24 hours.

Section 11: Detailed mathematical model for voltage generation of the lateral PNG built on a single PMN-PT nanobelt

Since the FIB-cut PMN-PT nanobelt was deposited onto the flexible polyimide (PI) substrate, fixed by silver paste on both ends and capped with a thin layer of polydimethylsiloxane (PDMS), the whole system can be regarded as a compact,

unidirectiontal nanobelt-enforced entity. The modulus E_{11} of the whole composite can be calculated as following:

$$E_{11} = E_{PMNPT}V_{PMNPT} + E_{PI}V_{PI} + E_{PDMS}\,(1 - V_{PMNPT} - V_{PI}) \quad (8)$$

Where E_{PMNPT}(18Gpa)[15], E_{PI} (2.5 GPa)[38] and E_{PDMS} (500 KPa)[39] are the Young's moduli of PMN-PT nanobelt, PI substrate, and PDMS. V_{PMNPT} and V_{PI} are the concentrations of PMN-PT nanobelt and PI substrate:

$$V_{PMNPT} = \frac{A_{PMNPT}}{A_{Total}} \quad (9)$$

$$V_{PI} = \frac{A_{PI}}{A_{Total}} \quad (10)$$

Where A is net cross-sectional area for PMN-PT nanobelt, PI substrate and the whole composite. The major Poisson's ratio can be given as

$$\nu_{12} = \nu_{13} = \nu_{PMNPT}V_{PMNPT} + \nu_{PI}V_{PI} + \nu_{PDMS}\,(1 - V_{PMNPT} - V_{PI}) \quad (11)$$

Where ν_{PMNPT} (0.37)[40], ν_{PI} (0.34)[41] and ν_{PDMS} (0.5)[42] are the Poisson's ratios of PMN-PT nanobelt, PI substrate and PDMS, respectively. The strain along the axial direction ($[001]_c$) of the PMNPT nanobelt can be obtained as:

$$\varepsilon_{xx} = \frac{\sigma_{xx}}{E_{11}} - \frac{\sigma_{yy}}{E_{11}}\nu_{12} - \frac{\sigma_{zz}}{E_{11}}\nu_{13} \quad (12)$$

where σ_{xx} , σ_{yy} , and σ_{zz} are the stresses of x (length), y (width) and z (height) directions. From the above equation, the stress function along the axial direction ($[001]_c$) of the PMNPT nanobelt can be expressed as:

$$\sigma(l) = E_{PMNPT}\varepsilon_{xx} = E_{PMNPT}\,(\frac{\sigma_{xx}}{E_{11}} - \frac{\sigma_{yy}}{E_{11}}\nu_{12} - \frac{\sigma_{zz}}{E_{11}}\nu_{13}) \quad (13)$$

Since the potential generated from the PMN-PT nanobelt between the two electrodes is given by:

$$\Delta V = \int_0^l g_{33}\,\sigma(l)dl \quad (14)$$

Where l is the length of the nanobelt across two electrodes, and g_{33} is the piezoelectric voltage constant (38.8 x 10^{-3} V m/N)[43]. The output voltage can be written as:

$$\Delta V = \int_0^l g_{33}\, E_{PMNPT}\, (\frac{\sigma_{xx}}{E_{11}} - \frac{\sigma_{yy}}{E_{11}} \nu_{12} - \frac{\sigma_{zz}}{E_{11}} \nu_{13}) dl \quad (15)$$

Section 12: Supplementary References

1 Xu, S. *et al.* Flexible Piezoelectric PMN–PT Nanowire-Based Nanocomposite and Device. *Nano Lett.* **13**, 2393-2398 (2013).

2 Chen, X., Xu, S., Yao, N. & Shi, Y. 1.6 V Nanogenerator for Mechanical Energy Harvesting Using PZT Nanofibers. *Nano Lett.* **10**, 2133-2137 (2010).

3 Xu, S., Poirier, G. & Yao, N. PMN-PT Nanowires with a Very High Piezoelectric Constant. *Nano Lett.* **12**, 2238-2242 (2012).

4 Wang, Z., Hu, J., Suryavanshi, A. P., Yum, K. & Yu, M.-F. Voltage Generation from Individual $BaTiO_3$ Nanowires under Periodic Tensile Mechanical Load. *Nano Lett.* **7**, 2966-2969 (2007).

5 Xu, S. *et al.* Self-powered nanowire devices. *Nat Nano* **5**, 366-373 (2010).

6 Chang, C., Tran, V. H., Wang, J., Fuh, Y.-K. & Lin, L. Direct-Write Piezoelectric Polymeric Nanogenerator with High Energy Conversion Efficiency. *Nano Lett.* **10**, 726-731 (2010).

7 Wang, X., Song, J., Liu, J. & Wang, Z. L. Direct-Current Nanogenerator Driven by Ultrasonic Waves. *Science* **316**, 102-105 (2007).

8 Sun, C., Shi, J. & Wang, X. Fundamental study of mechanical energy harvesting using piezoelectric nanostructures. *J.Appl. Phys* **108**, 034309 (2010).

9 Donelan, J. M. *et al.* Biomechanical Energy Harvesting: Generating Electricity During Walking with Minimal User Effort. *Science* **319**, 807-810 (2008).

10 Kuo, A. D. Harvesting Energy by Improving the Economy of Human Walking. *Science* **309**, 1686-1687 (2005).

11 Yang, R., Qin, Y., Li, C., Zhu, G. & Wang, Z. L. Converting Biomechanical Energy into Electricity by a Muscle-Movement-Driven Nanogenerator. *Nano Lett.* **9**, 1201-1205 (2009).

12 Wang, Z. L. & Song, J. Piezoelectric Nanogenerators Based on Zinc Oxide Nanowire Arrays. *Science* **312**, 242-246 (2006).

13 Yang, R., Qin, Y., Dai, L. & Wang, Z. L. Power generation with laterally packaged piezoelectric fine wires. *Nat Nano* **4**, 34-39 (2009).

14 Park, S. E. & Shrout, T. R. Ultrahigh strain and piezoelectric behavior in relaxor based ferroelectric single crystals. *J.Appl. Phys* **82**, 1804-1811 (1997).

15 Ewart, L. M. *et al. Sixteenth IEEE International Symposium on the Applications of Ferroelectrics, Vols 1 and 2,* 550-553 (2007).

16 Viehland, D. & Li, J.-F. Young's modulus and hysteretic losses of $0.7Pb(Mg_{1/3}Nb_{2/3})O_3$-$0.3PbTiO_3$: single versus polycrystalline forms. *J.Appl. Phys* **94**, 7719-7722 (2003).

17 Fu, H. & Cohen, R. E. Polarization rotation mechanism for ultrahigh electromechanical response in single-crystal piezoelectrics. *Nature* **403**, 281-283 (2000).

18 Berlincourt, D. & Jaffe, H. Elastic and Piezoelectric Coefficients of Single-Crystal Barium Titanate. *Phys. Rev.* **111**, 143-148 (1958).

19 Zhao Deng, Y. D., Wen Chen, Xinmei Pei, Jihong Liao. Synthesis and Characterization of Bowl-Like Single-Crystalline $BaTiO_3$ Nanoparticles. *Nanoscale Res. Lett.* **5**, 1217-1221 (2010).
20 Ke, T.-Y. *et al.* Sodium Niobate Nanowire and Its Piezoelectricity. *J. Phys. Chem C* **112**, 8827-8831 (2008).
21 Agrawal, R., Peng, B., Gdoutos, E. E. & Espinosa, H. D. Elasticity Size Effects in ZnO Nanowires−A Combined Experimental-Computational Approach. *Nano Lett.* **8**, 3668-3674 (2008).
22 Bernal, R. A. *et al.* Effect of Growth Orientation and Diameter on the Elasticity of GaN Nanowires. A Combined in Situ TEM and Atomistic Modeling Investigation. *Nano Lett.* **11**, 548-555 (2011).
23 Hoffmann, S. *et al.* Fracture strength and Young's modulus of ZnO nanowires. *Nanotechnology* **18**, 205503 (2007).
24 Saraf, G., Lu, Y. & Siegrist, T. In-plane anisotropic strain in a-ZnO films grown on r-sapphire substrates. *Appl. Phys. Lett* **93**, 041903 (2008).
25 Cohen-Tanugi, D., Akey, A. & Yao, N. Ultralow Superharmonic Resonance for Functional Nanowires. *Nano Lett.* **10**, 852-859 (2010).
26 Baek, S.-H., Rzchowski, M. S. & Aksyuk, V. A. Giant piezoelectricity in PMN-PT thin films: Beyond PZT. *MRS Bulletin* **37**, 1022-1029 (2012).
27 Wang, X., Liu, J., Song, J. & Wang, Z. L. Integrated Nanogenerators in Biofluid. *Nano Lett.* **7**, 2475-2479 (2007).
28 Kiat, J.-M. *et al.* Monoclinic structure of unpoled morphotropic high piezoelectric PMN-PT and PZN-PT compounds. *Phys. Rev. B* **65**, 064106 (2002).
29 Park, S.-E. & Shrout, T. R. *IEEE Ultrasonics Symposium Proceedings* 935-942 vol.932*,* (1996).
30 Wang, F., Luo, L., Zhou, D., Zhao, X. & Luo, H. Complete set of elastic, dielectric, and piezoelectric constants of orthorhombic $0.71Pb(Mg_{1/3}Nb_{2/3})O_3$-$0.29PbTiO_3$ single crystal. *Appl. Phys. Lett* **90** (2007).
31 Zhang, R., Jiang, B. & Cao, W. W. Single-domain properties of $0.67Pb(Mg_{1/3}Nb_{2/3})O_3$-$0.33PbTiO_3$ single crystals under electric field bias. *Appl. Phys. Lett* **82**, 787-789 (2003).
32 Viehland, D., Li, J. F. & Amin, A. Electromechanical and elastic isotropy in the (011) plane of $0.7Pb(Mg_{1/3}Nb_{2/3})O_3$-$0.3PbTiO_3$ crystals: Inhomogeneous shearing of polarization. *J.Appl. Phys* **92**, 3985-3989 (2002).
33 Zhao, M.-H., Wang, Z.-L. & Mao, S. X. Piezoelectric Characterization of Individual Zinc Oxide Nanobelt Probed by Piezoresponse Force Microscope. *Nano Lett.* **4**, 587-590 (2004).
34 Qi, Y. *et al.* Enhanced Piezoelectricity and Stretchability in Energy Harvesting Devices Fabricated from Buckled PZT Ribbons. *Nano Lett.* **11**, 1331-1336 (2011).
35 Wang, J. *et al.* Piezoresponse force microscopy on doubly clamped $KNbO_3$ nanowires. *Appl. Phys. Lett* **93**, 223101 (2008).
36 Güthner, P. & Dransfeld, K. Local poling of ferroelectric polymers by scanning force microscopy. *Appl. Phys. Lett* **61**, 1137-1139 (1992).
37 Wang, Z., Suryavanshi, A. P. & Yu, M.-F. Ferroelectric and piezoelectric behaviors of individual single crystalline $BaTiO_3$ nanowire under direct axial electric biasing. *Appl. Phys. Lett* **89**, 082903, (2006).
38 DuPont. DuPont™ Kapton® HN polyimide filmTechnical Data Sheet. (2011).
39 PDMS (polydimethylsiloxane) Material Property Database.
40 Nagakalyan, S., Raghukumar, B. & Abhilash, K. V. Comparative study of piezoelectric materials for vibration energy harvesting. *IJRAME* **1**, 160-166 (2013).
41 Bauer, C. L. & Farris, R. J. Determination of poisson's ratio for polyimide films. *Polym. Eng. Sci.* **29**, 1107-1110 (1989).

42 Pritchard, R. H., Lava, P., Debruyne, D. & Terentjev, E. M. Precise determination of the Poisson ratio in soft materials with 2D digital image correlation. *Soft Matter* **9**, 6037-6045 (2013).

43 Zhang, R., Jiang, B. & Cao, W. W. Elastic, piezoelectric, and dielectric properties of multidomain 0.67Pb($Mg_{1/3}Nb_{2/3}$)O_3-0.33PbTiO_3 single crystals. *J. Appl. Phys* **90**, 3471-3475 (2001).
